# Deep Eutectic Solvent-Assisted Synthesis of Ni–Graphene Composite Supported on Screen-Printed Electrodes for Biogenic Amine Detection

**DOI:** 10.3390/ma18020425

**Published:** 2025-01-17

**Authors:** Aleksandra Levshakova, Maria Kaneva, Ruzanna Ninayan, Evgenii Borisov, Evgenii Satymov, Alexander Shmalko, Lev Logunov, Aleksandr Kuchmizhak, Yuri N. Kulchin, Alina Manshina, Evgeniia Khairullina

**Affiliations:** 1 Institute of Chemistry, St. Petersburg State University, 199034 St. Petersburg, Russia; sashkeens@gmail.com (A.L.); skt94@bk.ru (M.K.); st098468@student.spbu.ru (R.N.); set167@gmail.com (E.S.); 2 Center for Optical and Laser Materials Research, St. Petersburg University, 199034 St. Petersburg, Russia; eugene.borisov@spbu.ru; 3 Nanotechnology Research and Education Centre RAS, Saint Petersburg Academic University, 194021 St. Petersburg, Russia; sanya050199@gmail.com; 4 School of Physics and Engineering, ITMO University, 191002 St. Petersburg, Russia; lev.logunov@metalab.ifmo.ru; 5 Institute of Automation and Control Processes, Far Eastern Branch, Russian Academy of Sciences, 690041 Vladivostok, Russia; alex.iacp.dvo@mail.ru (A.K.); kulchin@iacp.dvo.ru (Y.N.K.); 6 Polytechnic Institute, Far Eastern Federal University, 690090 Vladivostok, Russia

**Keywords:** deep eutectic solvent, laser fabrication, nickel–graphene composites, electrochemical sensor, biogenic amine, dopamine

## Abstract

Deep eutectic solvents (DES) have emerged as versatile, sustainable media for the synthesis of nanomaterials due to their low toxicity, tunability, and biocompatibility. This study develops a one-step method to modify commercially available screen-printed electrodes (SPE) using laser-induced pyrolysis of DES, consisting of choline chloride and tartaric acid with dissolved nickel acetate and dispersed graphene. The electrodes were patterned using a 532 nm continuous-wave laser for the in situ formation of Ni nanoparticles decorated on graphene sheets directly on the SPE surface (Ni-G/SPE). The synthesis parameters, specifically laser power and graphene concentration, were optimized using the Nelder–Mead method to produce modified Ni-G/SPEs with maximized electrochemical response to dopamine. Electrochemical characterization of the developed sensor by differential pulse voltammetry revealed its broad linear detection range from 0.25 to 100 μM and high sensitivity with a low detection limit of 0.095 μM. These results highlight the potential of laser-assisted DES synthesis to advance electrochemical sensing technologies, particularly for the detection of biogenic amines.

## 1. Introduction

Deep eutectic solvents (DES) are formed by mixing two main components: a hydrogen bond donor (HBD) and a hydrogen bond acceptor (HBA), which interact through extensive hydrogen bonding to form a eutectic mixture with a melting point significantly lower than that of the individual components [1,2]. The beneficial properties of DES, including sustainability, low toxicity, tunability, minimal vapor pressure, and the ability to dissolve a wide range of chemicals, have gained significant recognition in both scientific and industrial fields [3,4]. DES can provide cost-effective, less labor-intensive, environmentally friendly, and safe alternatives that are in line with the United Nations Sustainable Development Goals [5]. However, along with the benefits of DES, there are also challenges. Some DESs have high viscosity, which can make it difficult to apply a uniform film to substrates for laser processing and can affect mass transport and reaction kinetics [6]. In addition, certain DESs can degrade over time, especially when exposed to moisture or temperature changes, affecting their performance. Therefore, careful selection of the DES composition is critical to address these issues.

Due to the aforementioned advantages, DES has shown considerable potential as an effective medium for the production of a wide range of nanomaterials. This has been demonstrated in organic synthesis for the production of valuable organic compounds [7], as well as in the fabrication of inorganic nanomaterials and advanced carbon-based materials [8,9]. The successful synthesis of noble metal nanoparticles (NPs) has been performed, with DES-mediated processes yielding materials with enhanced plasmonic and catalytic properties [10,11]. Beyond noble metals, the synthesis of transition metal sulfides, oxides, nitride, and phosphates has also been effectively carried out in DES, extending its applicability to a wider range of materials [8,12,13,14,15,16,17,18]. The most extensively studied technique for creating transition metal nanostructures in DES is electroplating. In this process, DES is used as an electrolyte medium, which not only enables the deposition of metal ions onto substrates but also significantly influences the morphology and properties of the deposited layers [19,20]. Despite these advances, the preparation of pure transition metal nanoparticles in DES media beyond electrochemical conditions remains challenging.

Laser synthesis has emerged as a promising alternative approach for the synthesis of nanoparticles in DES. In this method, the high temperatures generated in the focal spot of the laser beam are used to decompose the organic components of DES, resulting in the release of highly active species that facilitate the reduction in metal ions and the subsequent formation of metallic nanoparticles. In the context of laser processing, a key advantage of using DES is that reaction rates exceed those of traditional water-based laser synthesis methods by more than two orders of magnitude [21], leading to improved manufacturing efficiency and significant economic benefits. Previous studies using laser-induced pyrolysis of DES have primarily focused on continuous conductive metallic patterns on substrates [22]. Applications for the resulting metallic structures have been found in Radio Frequency Identification (RFID) tags, contacts for LED devices, and, among others, in electrochemical non-enzymatic sensors [23].

A major advantage of laser-assisted synthesis of materials for electrochemical sensing is the ability to precisely shape electrodes through the localized nature of the laser process [24]. This localization eliminates the need for surfactants and surface binders such as Nafion, which are typically required to stabilize the NPs and anchor them to electrode surfaces [25]. As a result, this method simplifies the manufacturing process and can lead to an increase in the purity of the synthesized nanomaterials. Significant potential for the detection of a wide range of analytes has been demonstrated by metal-based composites and advanced carbon materials synthesized using this technique [26], demonstrating the validity for further expansion of target analytes, including biogenic amines (BAs). Biosynthesized in microorganisms, plants, and animals, BAs play a crucial role in various biological processes and are classified into endogenous and exogenous groups [27]. Monitoring of BA levels is essential in areas such as food safety and health care [28,29,30,31]. Dopamine is crucial for many bodily functions, but its abnormal levels can lead to a number of health risks. These include increased susceptibility to neurological and psychotic disorders, the development of addictive behaviors, mood disorders, and cardiovascular problems [32].

**Figure 1:**
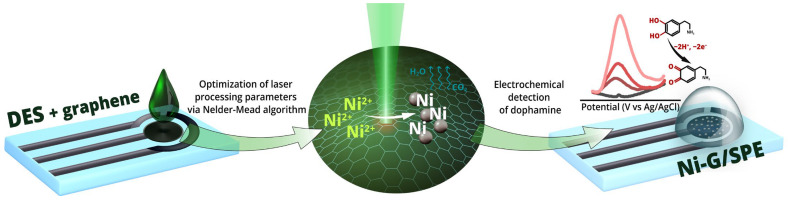
Electrode fabrication process and electrochemical testing.

In this study, the feasibility of a one-step modification of commercially available screen-printed electrodes (SPE) via laser-induced synthesis in DES is explored. Laser-assisted pyrolysis allows for in situ modification, resulting in more uniform distribution and improved adhesion of the modified layer to the electrode surface compared to traditional techniques such as drop casting [33]. This approach effectively addresses critical challenges in electrochemical sensors, particularly in terms of reproducibility and reliability. Nickel nanoparticles synthesized by this method exhibit excellent electrocatalytic activity in the electrooxidation of dopamine-to-dopamine quinone [34] while providing a cost-effective alternative to noble metals. Furthermore, the incorporation of graphene into the composite system is expected to improve the overall performance of the synthesized electrode by exploiting the synergistic effects of different nanomaterials [35]. The initial DES composition was adapted from our previous works, which were aimed at the synthesis of conductive continuous metal patterns [23,36]. The DES, composed of choline chloride (ChCl) and tartaric acid with dissolved nickel acetate (the molar ratio of these components was maintained at 1:1:1) and dispersed graphene, was drop-cast onto the surface of SPE and scribed using a 532 nm continuous-wave laser. The fabrication scheme for Ni-G/SPE is shown in Figure 1. The experimental parameters were optimized using the Nelder–Mead method, which significantly reduced both time and consumable costs, thus supporting the sustainability of the research. The sensor activity of this composite toward dopamine, as a model biogenic amine, was investigated. The analytical characteristics were evaluated using voltammetric techniques, including cyclic voltammetry (CV) and differential pulse voltammetry (DPV). The ability of the sensor to detect dopamine over a broad linear range with high sensitivity and low detection limits is demonstrated by the study, proving the potential of laser-assisted DES synthesis in the development of advanced electrochemical sensors.

## 2. Materials and Methods

### 2.1. Materials

Nickel (II) acetate tetrahydrate (NiAc_2_·4H_2_O), choline chloride (C_5_H_14_ClNO), tartaric acid (C_4_H_6_O_6_), sodium dihydrogen phosphate dihydrate (NaH_2_PO_4_·2H_2_O), disodium hydrogen phosphate dodecahydrate (Na_2_HPO_4_·12H_2_O), and dopamine (C_8_H_11_NO_2_) were purchased from Thermo Scientific (Fair Lawn, NJ, USA). Graphene dispersion in water (0.5 mg/mL) was purchased from XFNano (Nanjing City, China). All reagents were analytical grade and used without further purification. Ultrapure Milli-Q water was used for all experiments (18.2 MΩ cm).

### 2.2. Preparation of Deep Eutectic Solvent

Precisely measured amounts of choline chloride, an organic acid, and metal salt were combined to prepare a deep eutectic solvent. The molar ratio of these components was maintained at 1:1:1. The graphene dispersion was then added to the DES at different concentrations (3–50 wt%). The mixture was placed in sealed vials and heated at 110 °C until fully melted. The molten mixture was then transferred to a heated magnetic stirrer and stirred at 120–140 °C until a homogeneous, viscous liquid formed.

### 2.3. Electrode Fabrication

Electrodes were fabricated using a CW Nd:YAG laser with a wavelength of 532 nm and a maximum power output of 2 W. Commercially available SPEs (Poten Inc., Qingdao, China) served as conductive substrates for the application of the DES. A 5 μL volume of DES was uniformly applied to the working electrode of SPE using the direct transfer method. The modified electrodes were dried on a hot plate at 50 °C for 15 min. Subsequently, the electrode surface was scribed with a focused laser beam (NA = 0.4). The laser output power varied between 100 and 1200 mW, while the scanning speed was maintained at a constant 1.4 mm/s. An XYZ mechanical stage controlled the substrate’s movement during the laser-writing process, ensuring precise patterning.

### 2.4. Nelder–Mead Algorithm

**Figure 2:**
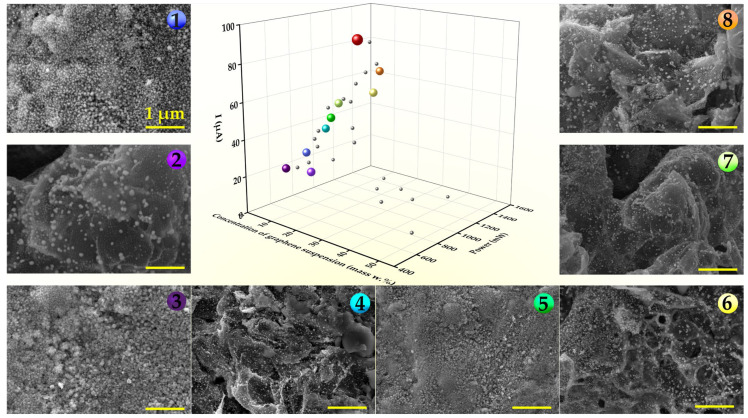
Nelder–Mead optimization of the Ni-G/SPE electrode electrochemical performance (maximal current) through variation in laser power and graphene concentration. Representative SEM images of the Ni-G/SPE samples synthesized in simplex vertices (differently colored points 1–8 on the central graph) at different values of laser power and concentration of graphene dispersion in DES.

The Nelder–Mead algorithm was used to optimize laser power and graphene dispersion concentration in DES. The primary performance metric for the efficiency of the electrode synthesis was its electrochemical response to dopamine. The optimization process began with the selection of three initial points in the parameter space, forming a triangular configuration (or simplex) (points 1–3 in Figure 2). The electrochemical response to dopamine was evaluated at each vertex, and the point exhibiting the highest response was identified. The algorithm iteratively performed a series of operations—contraction, expansion, and shrinkage of the triangle—to find the maxima of the electrochemical signal. The process continued until no further improvements in the response value were observed. A more detailed description of the Nelder–Mead method is provided in the Supplementary Materials.

### 2.5. Characterization Techniques

The electrode’s morphology and elemental composition were examined via scanning electron microscopy (SEM) at an acceleration voltage rate of 20 eV on a Zeiss Merlin system (Carl Zeiss, Oberkochen, Germany) and an INCAx-act energy-dispersive X-ray spectrometer (EDX) (Oxford Instruments, Abingdon, UK). Characterization of the phase composition was carried out with X-ray diffraction (XRD) analysis using a Bruker D2 Phaser diffractometer with a LynxEye detector (Bruker-AXS, Karlsruhe, Germany). Raman spectroscopy was performed with a confocal Senterra spectrometer (Bruker, Billerica, MA, USA), utilizing a 532 nm solid-state laser at 10 mW power and a 50× objective, with spectra collected twice. X-ray photoelectron spectroscopy (XPS) was carried out using an ESCALAB 250Xi electron spectrometer (ThermoFisher Scientific, Waltham, MA, USA).

### 2.6. Electrochemical Measurements

The electrochemical analysis was tested with a standard three-electrode cell using a Corrtest CS300 potentiostat (Wuhan CorrTest Co., Ltd., Wuhan, China) at room temperature. The supporting electrolyte for the determination of DA was a phosphate-buffer solution (0.1 M PBS, pH = 7.0). SPE and Ni-G/SPE were employed as working, 3 M KClAg/AgCl (LLC. “Measuring Technology”, Moscow, Russia) as a reference, and a platinum foil as a counter electrode. Additionally, small-volume sample analysis was performed using an SPE counter (carbon ink) and an SPE quasi–pseudo-reference electrode (AgQRE).

Cyclic voltammetry (CV) and differential pulse voltammetry (DPV) were used as electrochemical characterization techniques to assess the performance of the obtained sensors. CV measurements were conducted from −0.1 V to 0.8 V (vs. Ag/AgCl) with a scan rate of 50 mV/s. DPV curves were recorded with optimal parameters (amplitude 0.05 V, inc. E 0.004 V, pulse width 0.05 s, pulse period 0.5 s). All raw data were processed using the Origin 2018 9.5 trial version software.

## 3. Results and Discussion

### 3.1. Fabrication and Characterization of Ni-G/SPE via Laser-Assisted Synthesis Using the Nelder–Mead Optimization Approach

The Nelder–Mead [34] method was used to optimize the laser fabrication process by systematically adjusting the synthesis parameters to achieve a high sensor response of the fabricated electrodes to the target analyte. The Nelder–Mead method works by maintaining a simplex—a geometric figure consisting of n + 1 vertices in an n-dimensional parameter space. Through iterative processes of reflection, expansion, contraction, and shrinkage, the algorithm explores the parameter space to find the extremum of the objective function, which in this case was the maximization of the electrode response in 0.1 M PBS containing 100 µM DA.

The optimized parameters were laser power and graphene (G) dispersion concentration in the DES. The laser power controls the temperature in the reaction zone at a given scanning speed, while the graphene dispersion concentration affects the composition of the final structure. Therefore, these two parameters are essential for the one-step fabrication of the Ni-G composite on the SPE surface and allow for effective tuning of the electrochemical properties of the electrodes. The feasible ranges for output laser power and graphene concentration were determined to be 500–1500 mW and 3–50 mass%, respectively. The parameter limits for laser power were chosen based on previous results for laser-assisted nickel patterning from DES [32]. In turn, the graphene concentration was chosen over a wide range to ensure comprehensive experimentation. The Nelder–Mead algorithm-based approach provides a means for interactive experimentation. Here, the functional properties of the material are monitored directly throughout the development of the synthesis methodology. This is in contrast to the trial-and-error method, where functional properties are typically investigated, and optimal synthesis conditions are determined only after numerous samples have been produced under varying conditions. Such an approach allows for a significant reduction in the number of required experiments for material optimization tailored to specific applications.

**Figure 3:**
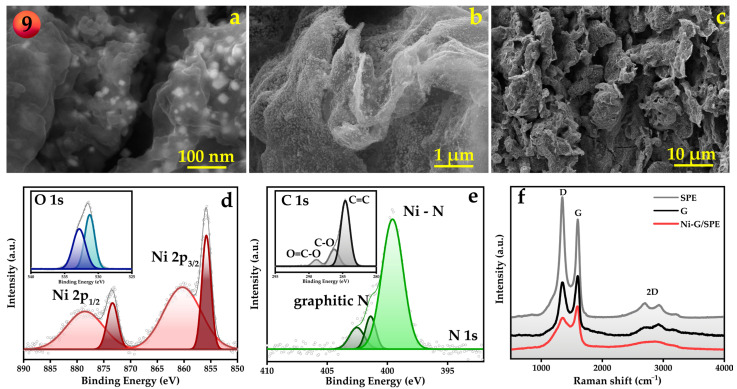
SEM images showing the morphology of the optimized Ni-G/SPE electrode (Sample 9) visualized at different magnifications (**a**–**c**), X-ray photoelectron spectra of the Ni 2p and O1s (**d**), N 1s and C 1s (**e**) regions as well as Raman spectra of SPE and Ni-G/SPE electrodes, graphene dispersion (**f**).

Based on the data on nickel pattern fabrication from DES [31,32], the starting point for Nelder–Mead optimization was chosen with a laser power of 540 mW and a graphene dispersion concentration of 10 mass%. Two additional points, located at a small distance along each of the parameter axes, were determined to form the initial simplex. At each point, laser synthesis of the electrode was performed, followed by measurement of the electrochemical response to dopamine. Table 1 presents the parameters used in the optimization process, including laser power (expressed as a percentage of the maximum), graphene concentration (in wt% relative to the initial DES mass), and the corresponding measured current values for each simplex coordinate. The table highlights key points such as the initial vertices (marked in blue), as well as significant outcomes like the median, reflection, expansion, and contraction points. The coordinate with the highest current response within each simplex is highlighted in green, while points, where laser-induced substrate damage was observed, are marked in red. The numbered points (1–9) correspond to the simplex vertices, visually represented by the colored spheres in Figure 2 and Figure 3.

The electrode fabricated under the optimized conditions (sample 9) was characterized by SEM, XRD, XPS, and Raman spectroscopy to investigate its morphological and structural properties. XRD patterns (Figure S1) show reflections corresponding to graphite from the original non-modified screen-printed electrode itself [37], as well as a broad peak corresponding to the polyethylene terephthalate (PET) substrate of the SPE [38]. In addition, reflections of metallic nickel were observed, confirming the reduction of nickel ions and the formation of metal nanoparticles due to laser-assisted pyrolysis. The diffraction peaks for Ni at 2θ values of 44.5° and 51.8° correspond to the characteristic (111) and (200) planes, which are indicative of face-centered cubic (fcc) structures [39,40].

The elemental composition and chemical states of the Ni-G/SPE were thoroughly investigated by XPS. The survey spectra (Figure S2) show the presence of Ni, C, N, and O. The high-resolution XPS spectrum of Ni 2p (Figure 3d) can be deconvoluted into two spin–orbit doublets and two shake-up satellites, with the main peaks observed at 873.4 and 855.8 eV, corresponding to a spin–orbit separation of 17.6 eV. According to the literature, these spectral features are indicative of nickel nanoparticles. The surface-sensitive nature of XPS, combined with the propensity of transition metal nanoparticles to form surface oxide layers and adsorb impurities, typically results in the presence of Ni^2+^ peaks in the spectra [39,41]. XPS analysis of oxygen reveals two distinct peaks, located at binding energies of 531.3 eV and 532.8 eV. The peak at 531.3 eV is typically associated with OH-groups and nickel–oxygen bonds, indicating the presence of nickel oxide or surface hydroxide species [42]. This suggests that some nickel nanoparticles on the composite surface have undergone partial oxidation. The second peak at 532.8 eV corresponds to the surface C-O bonds, which are characteristic of oxygen-containing functional groups on graphene sheets [43]. These groups may include epoxy or hydroxyl functionalities introduced during the laser-assisted synthesis process. The high-resolution XPS scan of N1s (Figure 3e) revealed the presence of three nitrogen species: the N–Ni peak centered at 399.6 eV [39,44], graphitic nitrogen at 401.4 eV [39,44], and a peak at 402.5 eV assigned to N2 trapped within the material during laser processing, as well as N-O surface contamination [45]. The formation of N–Ni and graphitic N species is highly favorable for electrochemical sensing applications and often results from high-temperature synthesis procedures [44,46]. The C 1s scan shows a spectrum typical of graphene structures and is consistent with the O 1s spectrum [47,48]. The main peak at 284.6 eV can be attributed to the sp^2^ carbon state, while the peaks at 286.3 eV and 288.9 eV indicate the presence of oxygen-containing functional groups.

The collected Raman spectra for the SPE, Ni-G/SPE, and graphene dispersion all exhibit the characteristic D and G bands typical of carbon-based materials (Figure 3f) [49,50]. Additionally, the 2D band is observed, which consists of several components. The multi-component nature of the 2D band can be attributed to oxidized forms of multilayer graphene and graphite [51,52]. The as-received SPE shows peaks specific to partially oxidized, highly oriented graphite. Considering the XRD data, a reasonable suggestion would be surface oxidation during manufacturing and storage, while the main component of the SPE is graphite. As long as the width of this 2D band corresponds to layer non-uniformity, i.e., the presence of lattice disorder and defects, it can be concluded that laser irradiation promotes the formation of defects in the graphene layers. Therefore, laser heating not only initiates the pyrolysis of DES but also modifies the carbon component of the composite. The increase in defect sites and the formation of oxygen-containing groups can lead to a higher number of electrochemically active sites of graphene, thereby accelerating electron transfer during the electrochemical oxidation of dihydroxybenzenes, including catechol [53]. Since the target biogenic amine, dopamine, is a catecholamine compound and its electrooxidation involves the oxidation of hydroxyl groups (Figure S3), it is reasonable to assume that the formation of more defective graphene may also promote its electrooxidation.

### 3.2. Electrochemical Characterization of Ni-G/SPE and Dopamine Detection

**Figure 4:**
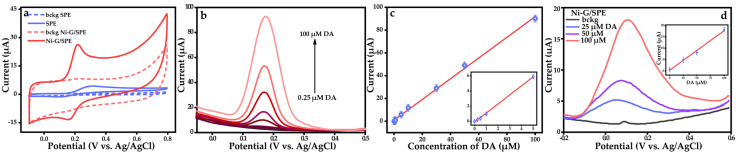
(**a**) CV curves of SPE and Ni-G/SPE in 0.1 M PBS without additives (dashed line) and in the presence of 50 μM DA (solid line), (**b**) DPV of the Ni-G/SPE electrode measured with the addition of various concentrations of DA, (**c**) Correlations of DA oxidation currents with respective concentrations, (**d**) DPV of the Ni-G/SPE in small volume samples (insert: Correlations of DA oxidation currents with respective concentrations).

Cyclic voltammetry measurements were performed in 0.1 M PBS supporting electrolyte in the presence of 50 µM DA to investigate the electrochemical behavior of the fabricated electrodes (Figure 4a). The Ni-G/SPE shows a well-defined oxidation peak corresponding to the oxidation of DA to dopamine quinone (DAQ) and a reduction peak corresponding to the reduction in DAQ back to DA (Figure S3). The Ni-G/SPE demonstrated a current response in a CV that was over 4.5 times greater than that of the bare SPE. Additional studies and discussions of the G/SPE and Ni/SPE are presented in the Supplementary Materials (Figure S4). The enhancement observed in the Ni-G/SPE, compared to other investigated electrodes, can be attributed to the incorporation of graphene on the electrode surface. This incorporation results in a composite with a larger active surface area than unmodified SPE, allowing a greater number of simultaneous electrochemical reactions per unit of geometric surface area. In addition, the decoration of graphene with Ni NPs leads to an increase in the electron transfer rate compared to the bare SPE, resulting in higher peak currents observed in CV. Faster electron transfer kinetics are also indicated by smaller potential differences between the anodic (Epa) and cathodic (Epc) peaks for the Ni-G/SPE. The smaller potential difference suggests that redox reactions on the modified electrode are more reversible.

Differential pulse voltammetry was used for DA analysis because of its high sensitivity (Figure 4b and Figure S5). The anodic peak current (Ipa) dependence on DA concentration was linear in the range of 0.25 to 100 µM, indicating an analytical sensitivity of 0.91 µA µM^−1^ (Figure 4c). The limit of detection (LOD) was determined to be 0.095 µM based on S/N = 3, where S is the standard deviation of the blank response and N is the slope of the calibration curve. The analytical performance of the Ni-G/SPE for DA detection was listed along with sensors previously described in the literature [54,55,56,57,58,59,60,61]. The properties of the developed electrode are comparable with many existing analogs (Table S1). However, it is worth highlighting the advantages of the Ni-G/SPE over similar electrodes, such as the precise modification of the electrode with localized Ni-G deposition on the SPE surface controlled by laser beam movement, while keeping the fabrication procedure rather simple. In addition, the well-known advantages of SPEs, including cost-effectiveness, versatility, scalability, and flexibility, make them indispensable tools in modern electrochemical research and commercial sensor development. In particular, these advantages highlight the superiority of electrochemical sensors over many other approaches, which, despite their own merits, often fall short in terms of portability and simplicity [62,63]. The developed modification strategy for SPEs significantly improves their electroanalytical performance in DA sensing. Using DES, this methodology facilitates the effective incorporation of advanced composite materials on the electrode surface, resulting in improved sensitivity, linear range, and LOD. Moreover, this approach is highly versatile and can be extended to the detection of a wide range of analytes as well as to the fabrication of other advanced composite electrodes.

In addition, the ability of Ni-G/SPE to detect DA in small-volume samples was demonstrated (Figure 4d). Aliquots of 50 µL containing three different concentrations of DA were used as model probes. The Ni-G/SPE electrode showed a prominent DPV response, and the peak current values fell within the linear regression, demonstrating the feasibility of the small-volume sensing. However, the electrochemical response differs from data collected in bulk cells. In bulk electrochemical cells, mass transport is primarily driven by both convection and diffusion. This combination ensures a consistent and uniform supply of analytes to the electrode surface, maintaining a stable electrical double layer and consistent charge transfer kinetics. In contrast, small-volume probe measurements rely primarily on diffusion for mass transport because convection is minimal or absent [61]. The limited volume and potential for evaporation in droplets affect the peak currents and the shape of the DPV curves. In this respect, and considering the promising initial results, further in-depth investigations are needed in the future to optimize the analytical protocol.

Further studies were aimed at investigating the selectivity, reproducibility, and repeatability of the analysis, as well as testing the electrodes for DA detection on real samples. The repeatability of the experiment was evaluated by eight successive measurements in a solution containing 50 µM DA. The relative standard deviation (RSD) of the peak current was 3.3%, indicating excellent repeatability of the electrode. Reproducibility was investigated based on measurements with three independently fabricated electrodes, and the RSD was found to be 4.5%. The stability test showed that after three months of storage under ambient conditions, the sensor signal maintained 95% of its initial value, indicating commendable stability. Such favorable analytical performance of the electrode can be attributed not only to the electrode composition but also to the optimized fabrication conditions that allow the modification of the SPE with Ni-G having good adhesion to the SPE surface. Adhesion was tested by performing a scotch tape test (Figure S6), confirming the robustness of the modified layer.

The most relevant coexisting substances in biological fluids are uric acid (UA) and ascorbic acid (AA), which are typically present at concentrations approximately 100 times higher than that of DA [62]. In this context, the selectivity of the fabricated sensor was tested by DPV in the presence of 50 µM DA and 100-fold excess concentrations of AA and UA, as well as other possible interferents and electrolytes. The results are presented in Table 2. All interferents tested, except UA, did not produce a significant analytical signal, and the signal deviation for DA did not exceed the calculated relative standard deviation for DA analyses without the interferents. For UA, an analytical signal was observed around 0.375 V. However, it does not affect the DA response, and the signal deviation for DA also did not exceed the RSD. The results obtained provide strong evidence for the excellent interference resistance of the developed electrode to the most abundant coexisting species.

The practical applicability of the Ni-G/SPE was confirmed by measurements on pharmaceutical samples of dopamine hydrochloride injections available at a local drugstore. The results are shown in Table 3. According to the manufacturer, the concentration of dopamine hydrochloride in the injection is 5 mg/mL (26 mM). Sample #1 was prepared by diluting a 9.6 µL aliquot of as-received injection solution in 50 mL 0.1 M PBS, suggesting the 5 µM of analyte in the final solution. Sample #2 was prepared similarly, except that the aliquot volume was increased to 38.4 µL. In addition, a protocol based on the standard addition method was also used (samples #3 and #4), where the dopamine hydrochloride injection solution in PBS was spiked with a DA solution of known concentration prepared in the laboratory. The developed sensor showed acceptable RSDs (N = 3) and recoveries, indicating satisfactory accuracy for real sample analyses.

## 4. Conclusions

This study demonstrates the feasibility and effectiveness of deep eutectic solvent-assisted laser synthesis for the fabrication of nickel-graphene-modified screen-printed electrodes for biogenic amine sensing. By using the Nelder–Mead optimization algorithm, key synthesis parameters—laser power, and graphene concentration—were fine-tuned to significantly improve the electrochemical performance of the sensor for dopamine detection. The Ni-G/SPE demonstrated excellent analytical performance with a wide linear range from 0.25 to 100 µM of DA, a low detection limit of 0.095 µM, and 0.91 µA µM^−1^ sensitivity. The practical applicability was confirmed by excellent reproducibility and the successful detection of dopamine in pharmaceutical samples, achieving high accuracy and reliable recovery rates. The combination of laser-assisted synthesis and mathematical optimization was proven to be a powerful strategy for the rapid development of high-performance electrochemical sensors. Due to the high degree of automation in the laser synthesis process using motorized stages and software control, the proposed fabrication approach does not require highly skilled personnel to perform. The high scanning speed of the laser beam, combined with a relatively simple synthesis protocol that eliminates the need for laborious pre- and post-treatment steps, significantly reduces the time required for electrode fabrication. Overall, the simplicity and low skill requirements of this technique, together with its time efficiency, make it of high practical value. Future research could focus on expanding this integrated technique to synthesize a broader range of materials.

## Figures and Tables

**Table 1:** Nelder–Mead optimization parameters.

Best	Good	Worst	x_m_	x_r_	x_e_	x_c_
10; 720 (1) I = 30.6	20; 540 (2) I = 27.9	10; 540 (3) I = 25.7	15; 630 I = 28.9	20; 720 (4) I = 53.0	25; 810 I = 39.9	12.5; 585 I = 26.2
20; 720 I = 52.9	10; 720 I = 30.6	20; 540 I = 27.9	15; 720 I = 44.2	10; 900 (5) I = 40.4	5; 1080 I = 16.9	17.5; 630 I = 42.6
20; 720 I = 52.9	10; 900 I = 40.43	10; 720 I = 30.6	15; 810 I = 55.2	20; 900 I = 58.4	25; 990 (6) I = 63.4	12.5; 765 I = 33.9
25; 990 I = 63.4	20; 720 I = 52.9	10; 900 I = 40.43	22.5; 855 I = 45.9	35; 810 I = 12.0	47.5; 765 I = 2.7	16.3; 880 (7) I = 56.8
25; 990 I = 63.4	16.3; 880 I = 56.8	20; 720 I = 52.9	20.6; 935 I = 67.7	21.3; 1150 (8) I = 71.6	21.9; 1360 I = 0	20.3; 825 I = 61.2
21.3; 1150 I = 71.6	25; 990 I = 63.4	16.3; 880 I = 56.8	23.1; 1070 I = 77.1	30; 1260 I = 0	36.9; 1360 I = 0	19.7; 975 (9) I = 90.1
19.7; 975 I = 90.1	21.3; 1150 I = 71.6	25; 990 I = 63.4	20.5; 1070 I = 88.0	15.9; 1260 I = 0	11.4; 1450 I = 0	22.7; 975 I = 73.7

**Table 2:** Influence of interference agents on the determination of 50 μM DA.

Interfering Agent	Fold Excess Concentration	Signal Change (%)
Uric acid	100	4.6
Ascorbic acid	100	2.7
Glucose	100	4.5
K^+^	30	2.1
Na^+^	30	1.7
Fe^3+^	30	2.4
Cl^−^	30	3.1
NO^3−^	30	1.9
SO_4_^2−^	30	2.3

**Table 3:** Determination of DA in real and spiked samples.

Sample No	DA			
	Added, µM	Found, µM	Recovery, %	RSD ^a^
1	5	4.9	96.3	3.7
2	20	20.1	101.5	1.7
3	15	15.1	102.2	2.8
4	30	30.2	102.5	3.1

^a^ Measurement values taken from three experiments.

## Data Availability

The original contributions presented in this study are included in the article and Supplementary Materials. Further inquiries can be directed to the corresponding authors.

## Supplementary Materials

The following supporting information can be downloaded at: https://www.mdpi.com/article/10.3390/ma18020425/s1, Figure S1. XRD spectra of SPE and Ni-G/SPE electrodes; Figure S2. The survey spectra of Ni-G/SPE electrode; Figure S3. Electrooxidation mechanism of dopamine; Figure S4. SEM images of (a) SPE, SPE modified with (b) graphene and (c) Ni NPs, (d) CV curves of G/SPE and Ni/SPE in 0.1 M PBS without additives (dashed line) and in the presence of 50 μM DA (solid line); Figure S5. DPV of the SPE electrode measured in 0.1 M PBS background solution and with the addition of various concentrations of DA; Figure S6. Peel test after 3 and 5 iterations. Table S1. Comparison of performance of Ni-G/SPE with other DA sensors described in the literature.
