# Supplementary material for: Deep Eutectic Solvent-Assisted Synthesis of Ni–Graphene Composite Supported on Screen-Printed Electrodes for Biogenic Amine Detection
Source: Materials (Basel). 2025 Jan 17;18(2):425. doi: 10.3390/ma18020425 (PMC11766956; doi:10.3390/ma18020425)
Supplement: Supplementary file 1 [file materials-18-00425-s001.zip › materials-3367972-supplementary.pdf]

materials

MDPI

Supplementary Materials

Deep Eutectic Solvent-Assisted Synthesis of Ni–Graphene Composite Supported on Screen-Printed Electrodes for Biogenic Amine Detection

Aleksandra Levshakova [1], Maria Kaneva [1], Ruzanna Ninayan [1], Evgenii Borisov [2], Evgenii Satymov [1], Alexander Shmalko [3], Lev Logunov [4], Aleksandr Kuchmizhak [5,6], Yuri N. Kulchin [5], Alina Manshina [1,*] and Evgeniia Khairullina [1,5,*]

[1] Institute of Chemistry, St. Petersburg State University, 199034 St. Petersburg, Russia; sashkeens@gmail.com (A.L.); skt94@bk.ru (M.K.); st098468@student.spbu.ru (R.N.); set167@gmail.com (E.S.)
[2] Center for Optical and Laser Materials Research, St. Petersburg University, 199034 St. Petersburg, Russia; eugene.borisov@spbu.ru
[3] Nanotechnology Research and Education Centre RAS, Saint Petersburg Academic University, 194021 St. Petersburg, Russia; sanya050199@gmail.com
[4] School of Physics and Engineering, ITMO University, 191002 St. Petersburg, Russia; lev.logunov@metalab.ifmo.ru
[5] Institute of Automation and Control Processes, Far Eastern Branch, Russian Academy of Sciences, 690041 Vladivostok, Russia; alex.iacp.dvo@mail.ru (A.K.); kulchin@iacp.dvo.ru (Y.N.K.)
[6] Polytechnic Institute, Far Eastern Federal University, 690090 Vladivostok, Russia
* Correspondence: a.manshina@spbu.ru (A.M.); e.khayrullina@spbu.ru (E.K.)

Description of the Nelder–Mead method

Optimization using the Nelder–Mead method helps to fine-tune parameters such as laser power and graphene suspension concentration to achieve the maximum electrode response for dopamine detection. This is achieved through successive steps of reflection, expansion, contraction, and shrinkage in the multidimensional parameter space. At each step, the method allows for moving the simplex vertices (synthesis parameters) toward those that yield better results, minimizing the response error.

Operations in the Nelder–Mead method:

Reflection:

$$x_{new}=x_{center}+\alpha(x_{center}-x_{worst}),$$

where x_{center} — the centroid of the remaining simplex vertices; x_{worst} — the worst vertex of the simplex (the vertex with the lowest response function value); α— the reflection coefficient (in our case α=1).

Expansion:

$$x_{new}=x_{center}+\gamma(x_{center}-x_{worst}),$$

where γ — the expansion coefficient (in our case γ=2).

Contraction:

$$x_{new}=x_{center}+\beta(x_{worst}-x_{center}),$$

where β — the contraction coefficient (in our case β=0.5).

Shrinkage: If none of the steps (reflection, expansion, contraction) result in an improvement, the simplex undergoes shrinkage. This means that all vertices except the best one are moved toward the best vertex:

$$x_{new} = x_{best} + \sigma(x_i - x_{best}),$$

where x_{best} — the best vertex of the simplex (the one with the highest response function value); x_i — the other simplex vertices (which are compressed toward x_{best}); σ — the shrinkage coefficient (in our case σ=0.5).

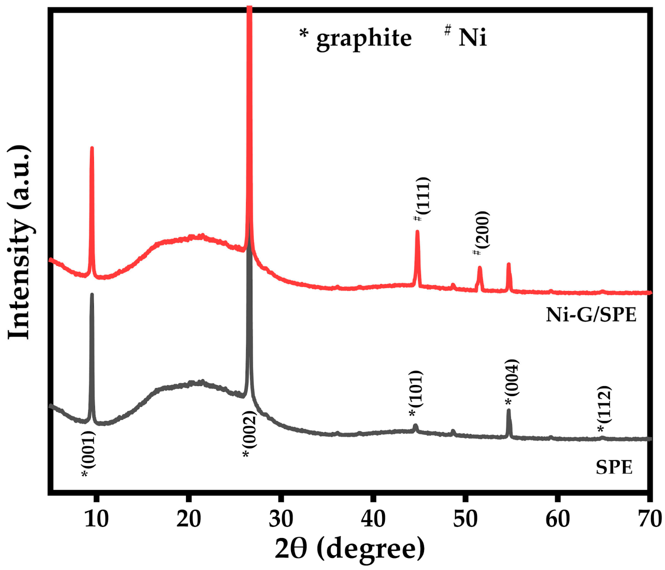

Figure S1. XRD spectra of SPE and Ni-G/SPE electrodes

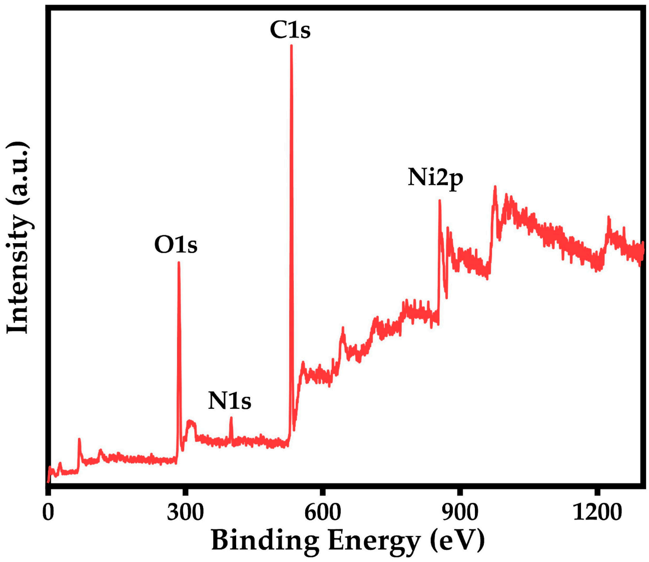

Figure S2. Survey XPS spectra of Ni-G/SPE electrode

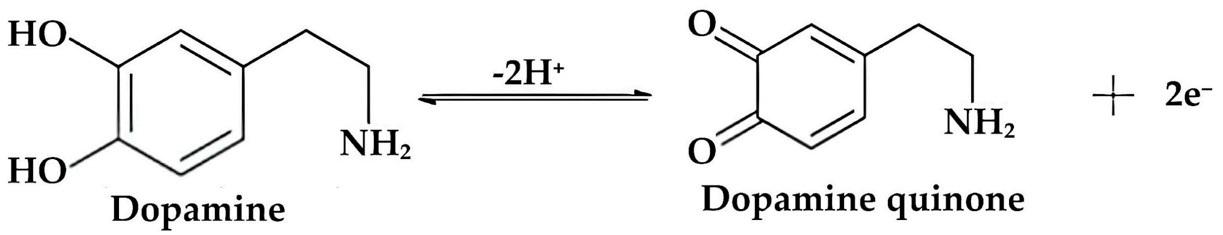

Figure S3. Electrooxidation mechanism of dopamine

Electrodes modified with Ni NPs by laser-assisted pyrolysis of DES without the addition of graphene, as well as electrodes modified with graphene, were fabricated. The latter was prepared by laser treatment of a drop-cast graphene dispersion under conditions optimized for the synthesis of the Ni-G composite. SEM images of unmodified SPE, graphene-modified SPE (G/SPE), and nickel-modified SPE (Ni/SPE) are shown in Figure S4. CV measurements of the G/SPE and Ni/SPE compared to the Ni-G/SPE showed that the Ni-G/SPE exhibited the highest response, confirming the presence of synergistic effects. Based on the collected data, one can tentatively conclude that the Ni-NPs facilitate a lower dopamine oxidation potential, while the graphene primarily contributes by providing a larger active surface area compared to unmodified SPE. However, these conclusions warrant further careful investigation to clarify the individual role of each component in the observed synergistic effect.

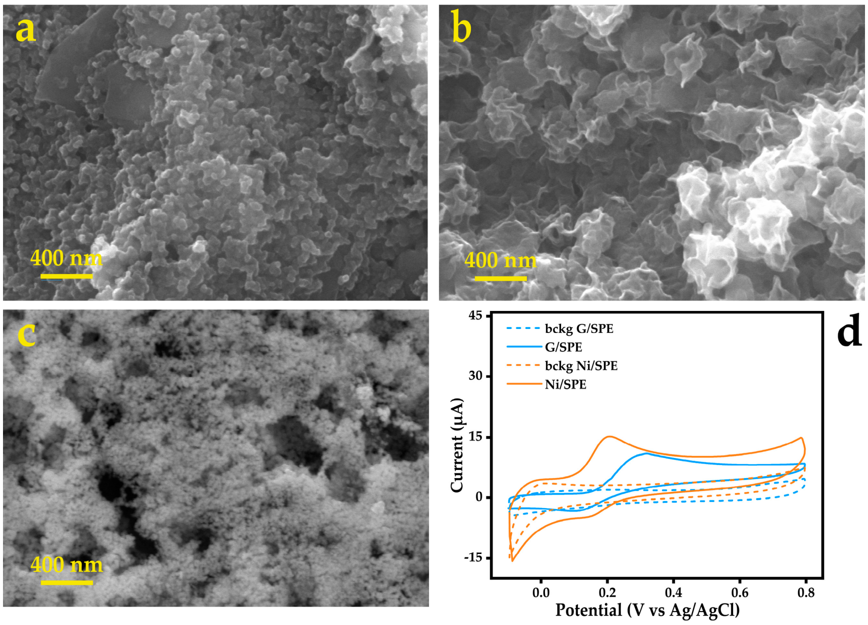

Figure S4. SEM images of (a) unmodified SPE, SPE modified with (b) graphene and (c) Ni NPs, (d) CV curves of G/SPE and Ni/SPE in 0.1 M PBS without additives (dashed line) and in the presence of 50 μM DA (solid line)

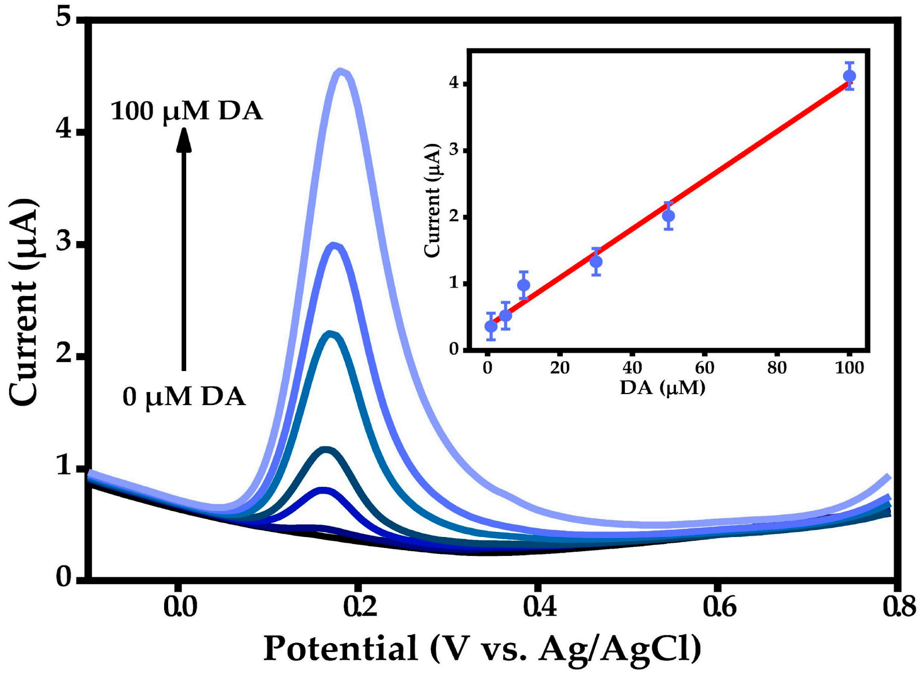

Figure S5. DPV of the SPE electrode measured in 0.1M PBS background solution and with the addition of various concentrations of DA

Table S1. Comparison of performance of Ni-G/SPE with other DA sensors described in the literature.

Composite	Synthesis method	Electrolyte	LOD, µM	Detection range, µM	Ref.
Ni/SPE	laser pyrolysis of DES	0.1 M PBS (pH = 7.0)	0.095	0.25 – 100	This work
Ni@N-doped C	calcination of covalent organic framework (COF)	0.1 M PBS (pH = 7.0)	0.009	0.027 – 70	[54]
NiO/CoO@PCNs/CNTs/ erGO	pyrolysis Ni/Co-MOF	0.1 M PBS (pH = 7.0)	0.045	0.10 – 22.0	[55]
Ni@CNF	wet impregnation method	0.1 M PBS (pH = 7.0)	0.030	0.1 – 10	[56]
GCE/EG-Ni-Au(NPs)	electrodeposition	0.1 M PBS (pH = 6.0)	0.100	0.2–1000	[57]
OMC/G/Ni	chemical vapor deposition and carbonization	0.1 M PBS (pH = 7.4)	0.019	0.05 – 58.75	[58]
Ni-Co-P NSs	hydrothermal method	0.1 M PBS (pH = 7.0)	0.016	0.3 – 50	[59]
GO-Ni3S2@GCE	hydrothermal technique, drop casting	0.1 M PBS (pH = 7.0)	0.012	2.5 – 270	[60]
GCox/nano-NiOx	electrodeposition, electrochemical oxidation	0.1 M PBS (pH = 6.0)	0.690	80.0-800	[61]

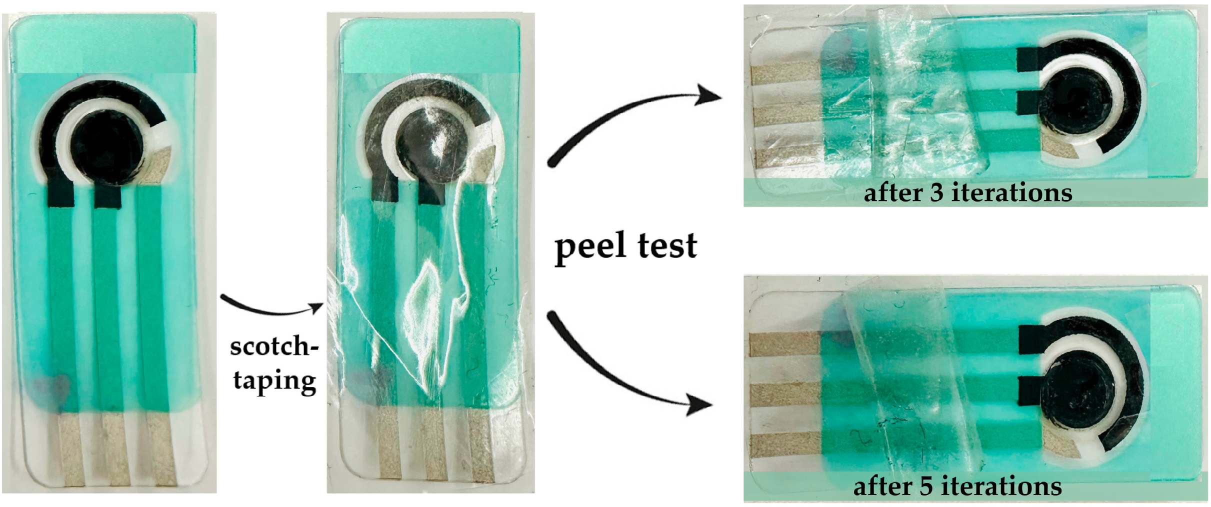

Figure S6. Peel test after three and five iterations
